# Detection of the spatial patterns of water storage variation over China in recent 70 years

**DOI:** 10.1038/s41598-017-06558-5

**Published:** 2017-07-25

**Authors:** Zheng Chen, Weiguo Jiang, Jianjun Wu, Kun Chen, Yue Deng, Kai Jia, Xinyu Mo

**Affiliations:** 1 Key Laboratory of Environmental Change and Natural Disaster, Beijing Normal University, Beijing, 100875 China; 2 Faculty of Geographical Science, Beijing Normal University, Beijing, 100875 China; 3 School of Resources and Environmental Engineering, Ludong University, Yantai, 264025 China; 4 State Key Laboratory of Remote Sensing Science, Institute of Remote Sensing and Digital Earth, Chinese Academy of Sciences, Beijing, 100101 China

## Abstract

Terrestrial water storage (TWS) variation is crucial for global hydrological cycles and water resources management under climatic changes. In the previous studies, changes in water storage of some part of China have been studied with GRACE data in recent ten years. However, the spatial pattern of changes in water storage over China may be different in a long period. Here, we aimed to present long-term spatial patterns of TWS over China between 1948 to 2015 by unique Global Land Data Assimilation System Version 2 data and identify possible factors to water storage changes. The results revealed that the inner-annual variations in TWS of China exhibited remarkable downward trends with decreased rate of 0.1 cm/yr. Meanwhile, we found that spatial patterns of TWS in China can be divided into three distinct sub-regions of TWS region with increased, TWS region with decreased, TWS region with insignificant variation. The Northeast had decreased trends (−0.05 cm/yr) due to climate change and anthropogenic activities. Urban expansion is a non-ignorable factor to TWS reduction in Jing-Jin-Ji region (r = 0.61); the west had increased from 1948 to 2015 (0.03 cm/yr) due to precipitation increased and recharge by glacier melt; the south had insignificant trends and TWS varied with precipitation (r = 0.78).

## Introduction

Terrestrial water storage (TWS) variation is crucial for global hydrological cycles and water resources management under climatic changes. However, TWS distributed unevenly in different regions and there would be serious losses as extreme droughts occurred. Therefore, it is necessary to detect spatial patterns of TWS, especially for China. Although there are several large rivers and vast amounts of wetlands in China, water shortage is persistent problem in some areas (e.g. Gansu, located in western China), because of certain climatic condition and the uneven distribution of water resources. In recent decades, several extreme droughts affected most parts of China^1, 2^, affecting many people and resulting in serious losses. The hydrological process and water resource management have been important topics of concern.

Ground measurement datasets derived from hydrologic stations could be used to estimate terrestrial water storage (TWS) variation with hydrological models. However, the datasets cannot meet the requirement of water storage change research at a large scale^3^. The Gravity Recovery and Climate Experiment (GRACE) satellite launched in 2002 has provided a new and effective method for water resource research^4^. It provides monthly change information about the mass distribution on the Earth’s surface^5^. Numerous researchers have acquired terrestrial water storage change information from GRACE measurements. Ndehendehe *et al*. (2016) successfully estimated TWS variations based on GRACE data in West Africa from 2002 to 2014^6^. Frappart *et al*. (2013) suggested that TWS variations estimated based on GRACE data could clearly exhibit the droughts and floods affected South America from 2003 to 2010^7^. However, the GRACE data spans only thirteen years and are not sufficient to investigate the temporal characteristics of water storage variation over a long period. Comparatively, the data derived from the Global Land Data Assimilation System version 2 (GLDAS-2) provide observations since 1948, a period of nearly 70 years. Meanwhile, GLDAS-2 at a resolution of 0.25° × 0.25° (Noah), is more spatially detailed than GRACE at a spatial resolution of 1° × 1° and WaterGAP WGHM2.2 at a spatial resolution of 0.5° × 0.5°^8^. Additionally, GLDAS is often used in the validation of GRACE measurements, and water storage changes derived from GLDAS are consistent with those estimated from GRACE in previous studies^9–11^. Nevertheless, Yang & Chen (2015) suggested that GLDAS is more sensitive to climate change than GRACE^5^.

In China, the terrestrial water storage varies regionally. Song *et al*. (2013) suggested that the total lake water storage of the Tibetan Plateau increased, whereas mass in southeastern Tibet and along the Himalayas decreased^12^. In the Badain Jaran Desert (western Inner Mongolia, China), both lake level and groundwater storage decreased from 2003 to 2009^13^. Huang *et al*. (2013) suggested that a significant decrease in TWS has occurred in the Yangtze River basin since 1998^14^. In the Tarim River basin (northwest China), TWS increased from 2003 to 2011 because of the recharge of snow melt^15^. TWS also corresponds to flooding and drought conditions^16, 17^. Long *et al*. (2014) estimated the TWS of the Yun-Gui Plateau from the 1980s to 2012, and the results showed that TWS anomalies correspond well to flood and drought events^18^. Although variations in TWS of different regions in China had been researched, little studies are related to TWS variation spatial pattern over China in a long term period. Thereafter, it is necessary to detect spatial patterns of water storage variation over China.

In this paper, we detected the spatial variation patterns of water storage over China for the period 1948–2015 at national and regional scales. We selected eight basins (Yangtze River Basin, Yellow River Basin, Heilongjiang River Basin, Liaohe River Basin, Haihe River Basin, Huaihe River Basin, Southeastern Rivers Basin and Pearl River Basin) and four regions (Gansu-Inner Mongolia, Xinjiang Province, Tibet-Qinghai Province and Yunnan Province) as the key regions to analyse the variation patterns at regional scales. This study aims to (1) detect the spatial pattern of TWS over China, (2) detect the regions with significant increases and decreases in TWS, and (3) identify the main factors influencing TWS variations in different regions.

## Results

### Water storage trend over China

**Figure 1:**
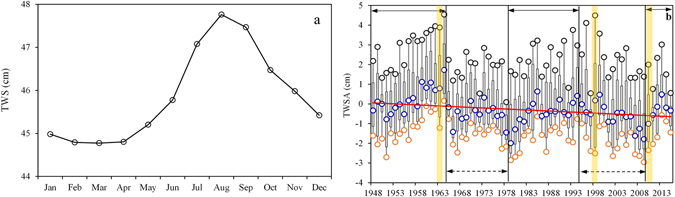
Inner-annual (**a**) and intra-annual (**b**) change trends of TWS from January 1948 to December 2015 (the grey symbol is the annual maximum value; the blue symbol is the annual median value; the orange symbol is the annual minimum value).

The inner-annual variation in TWS is significant, approximately 3 cm (Fig. 1a). TWS increases from January to August and decreases from August to December. It reaches a maximum of approximately 47.76 cm in August. TWS increases most rapidly from June to July, decreases most rapidly from September to October, and it has no obvious change from February to April.

Overall, the terrestrial water storage anomaly (TWSA) has decreased from 1948 to 2015 by approximately 0.1 cm/yr, especially in April to June and October to December (slope < −0.01), whereas water storage decreased insignificantly (slope > −0.01) in spring (from January to March) and summer (from July to September) (Fig. 1b and Fig. S2). From 1950s to 1965, TWSA increased in each month while TWSA decreased from 1960s to 1980. TWSA decreased slightly in Spring, Summer and Winter from 1990 to 2010. From 1980 to 2000, water storage increased significantly in August and September, about 4 cm. From 2010 to 2015, water storage increased in each month.

There are three increasing periods: 1948 to 1964, 1979 to 1994 and 2009 to 2015 (Table 1). From 1965 to 1978 and from 1995 to 2009, TWS tends to decrease (Table 1). In 1963 and1998, two extremely serious flood disasters occurred in Hebei province, Beijing, Tianjin, the Yangtze River basin and northern China due to heavy rainfall, thereby causing the maximum annual TWSA to reach peak values for the period of 1948 to 2015 (approximately 4 cm). A serious drought occurred in the southwestern region of China (Neimenggu province, northern, northeastern and southwestern China) in 2009, resulting in the minimum annual TWSA (approximately −3 cm).

**Table 1:** The increasing and decreasing periods of annual average TWSA between 1948 to 2015.

Increasing periods	Decreasing periods
1948–1964	1965–1978
1979–1994	1995–2009
2009–2015	

**Figure 2:**
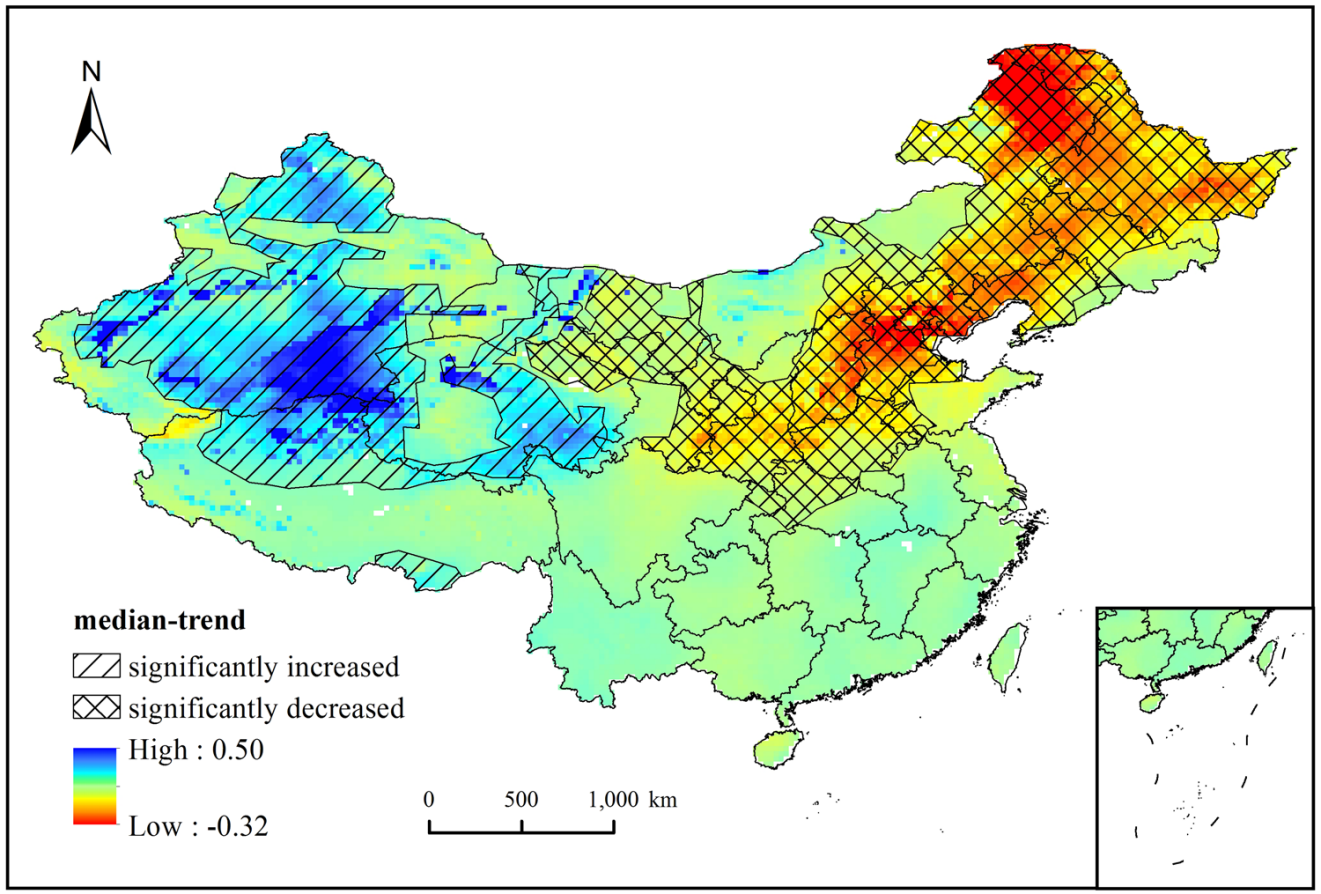
The spatial distribution of TWSA variation trends (*p* < 0.05). This map was created using ArcGIS 10.1 software, visit http://desktop.arcgis.com/en/ for more details.

Spatially, water storage decreased by 59.5% of China, and 55.4% of regions showed significant decreases (p < 0.05, including Ningxia, Gansu, Shaanxi, Shanxi, Henan, Beijing, Tianjin, Heibei, Liaoning, Jilin, Heilongjiang and the north of Neimenggu Province; Fig. 2). The depletion rate of water storage peaked in the north of Neimenggu, i.e., > 0.2 cm/yr. The water storage increased obviously in Xinjiang province and the Qinghai-Tibet plateau. The total increase area accounts for 40.5% of China, and 50.8% regions exhibited significant increases (Fig. 2), with a peak increase rate value of approximately 0.49 cm/yr.

### Spatial and temporal trend analysis of TWS in key zones

We selected eight basins (Yangtze River, Yellow River, Heilongjiang River, Liaohe River, Haihe River, Huaihe River, Southeastern Rivers and Zhujiang River) and four regions (Gansu-Inner Mongolia, Xinjiang Province, Tibet-Qinghai Province and Yunnan Province) as the key zones to study the spatial and temporal variation of TWS in different parts of China (Fig. S3). Generally, the TWS of southern China is more than that of northern China, similar to the spatial distribution of precipitation. The TWS of southeastern China is the highest (approximately 66.61 cm), and that of Xinjiang Province is the lowest (approximately 32.95 cm) (Table S1 and Fig. S4).

**Figure 3:**
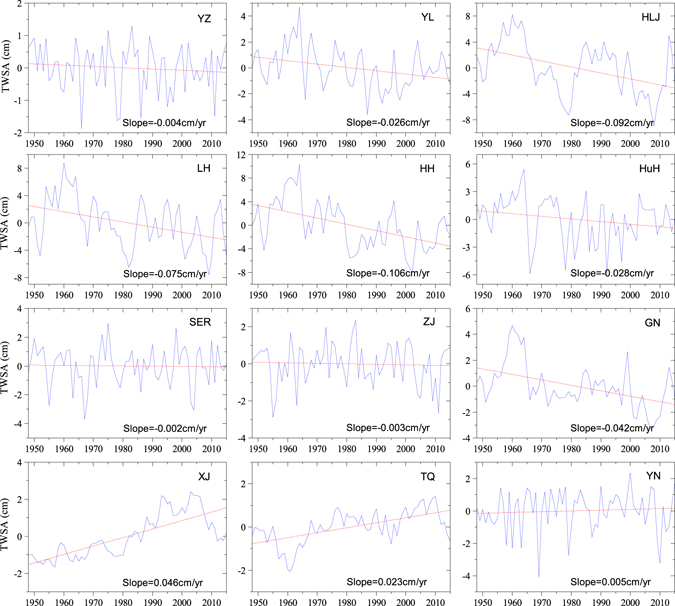
Long term changes of TWSA in key zones (YZ, YL, HLJ, LH, HH, HuH, SER, ZJ, GN, XJ, TQ and YN) from 1948 to 2015. This result was performed using Origin 9.0 software.

From 1948 to 2015, water storage tended to decrease in YL, HLJ, LH, HH, HuH and GN (−0.026~−0.106 cm/yr), whereas the variations of water storage showed increases in XJ and TQ, 0.023~0.046 cm/yr) (Fig. 3 and Table S2). In four other southern regions, no obvious changes are observed (0.002~0.005 cm/yr) (Fig. 3 and Table S2). The rate of water storage variation in XJ (approximately 0.046 cm/yr) is higher than that in TQ (approximately 0.023 cm/yr). In TQ, TWS rose from 1961 to 2010 by approximately 3.48 cm, decreased from 1948 to 1960 by approximately 1.58 cm, increased obviously over the period from 1948 to 2006 by approximately 3.17 cm/yr and decreased since 2007 by approximately 1.40 cm/yr in XJ. The rate of TWSA decrease in HH (approximately −0.106 cm/yr) was the highest among key zones with a decreasing tendency, and changes in TWSA showed obvious decreases in all decreasing regions from the 1960s to 1970s. In HLJ, the decreasing rate of TWSA was approximately −0.092 cm/yr from 1948 to 2015 and from 1960 to 1979, TWSA decreased by 15.51 cm. In addition, TWSA decreased by 10.73 cm and 6.26 cm in LH and HH from 1960 to 1979, respectively. Although there were no obvious changes, TWSA fluctuated frequently in the south of YZ and HuH. There are four obvious valley values in YZ around 1966, 1978, 1986 and 2011, and in HuH, values of TWS are frequently less than the annual average value, and there are three significant valley values around 1966, 1978 and 1992. In Yunnan province, TWS reached minimum values around 1969, 1979 and 2010. The low values may have been caused by serious drought events (Fig. S5).

**Figure 4:**
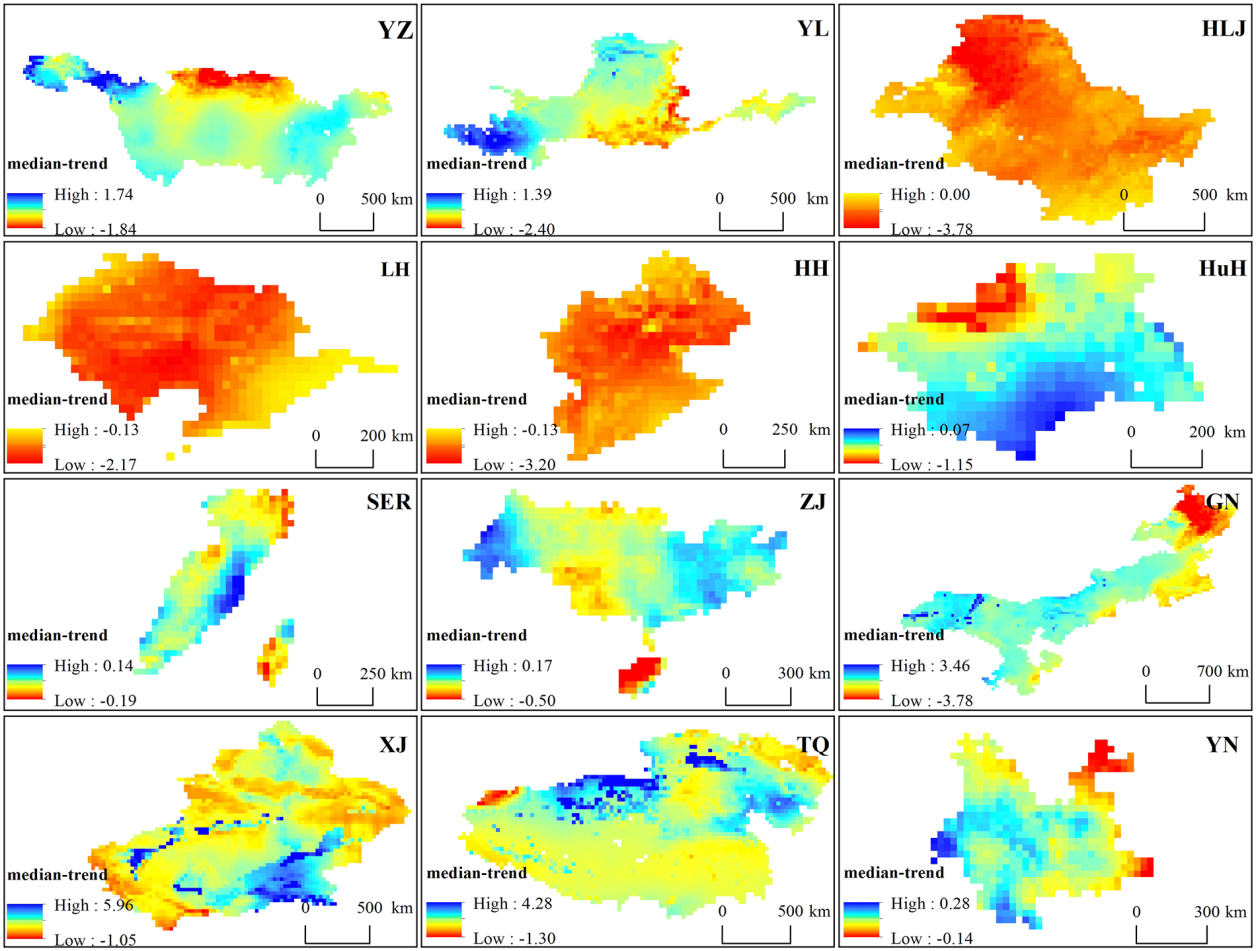
Spatial distribution of the annual average TWS change trend in each key zone. These results were performed by MATLAB 2013b and this map was created using ArcGIS 10.1 software, visit http://desktop.arcgis.com/en/ for more details.

We spatially calculated the TWS change trends in each key zone from 1948 to 2010 using the Theil-Sen median analysis method (Fig. 4). The variation ranges of SER, Yunnan and PL are the lowest, whereas those of GN, XJ and TQ are the largest. In the source region of YZ and YL (part of TQ), TWS shows an increasing tendency, whereas in northern YZ and the middle and lower reaches of YL, TWS tends to decrease in the period. In addition, TWS decreases in almost in the whole region of HLJ, LH, HH and HuH, especially in northern HLJ (the same region of northern GN) and the middle of LH and HH. In Hoh Xil (the junction of XJ and TQ), TWS increases obviously, whereas in the Tianshan mountains, TWS decreases in the study period. In YN, TWS tends to decrease only in the most eastern region.

**Figure 5:**
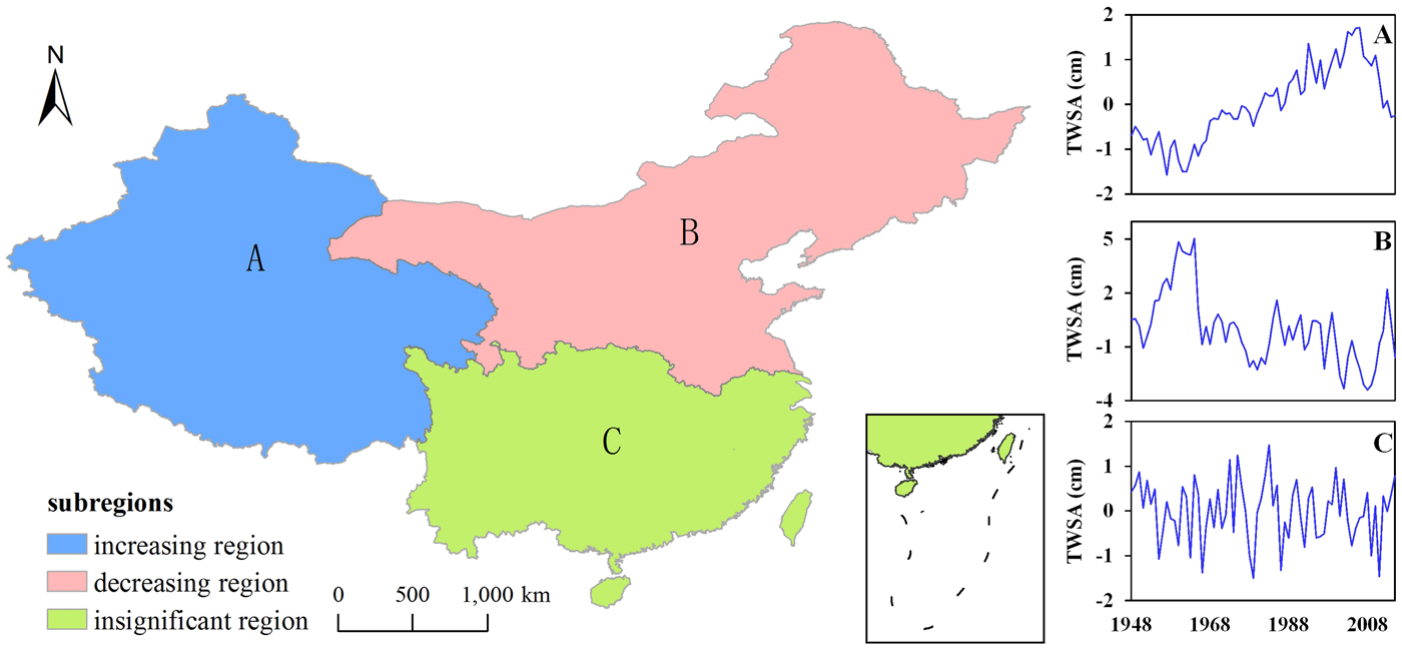
Spatial distributions of the TWS variation sub-regions: (**A**) increasing TWS region, (**B**) decreasing TWS region, and (**C**) the insignificant change region. This map was created using ArcGIS 10.1 software, visit http://desktop.arcgis.com/en/ for more details.

According to the spatial temporal variable characteristics shown in Figs 3 and 4, TWSA tends to increase in western China and decrease in northeastern China, whereas in southern China TWSA shows an insignificant tendency. Thus, we divided China into three sub-regions (Fig. 5): (1) the increasing TWS region (XJ and TQ); (2) the decreasing TWS region (YL, HLJ, LH, HH, HuH and GN); (3) the insignificant change region (YZ, SER, ZJ and YN).

## Discussion

**Figure 6:**
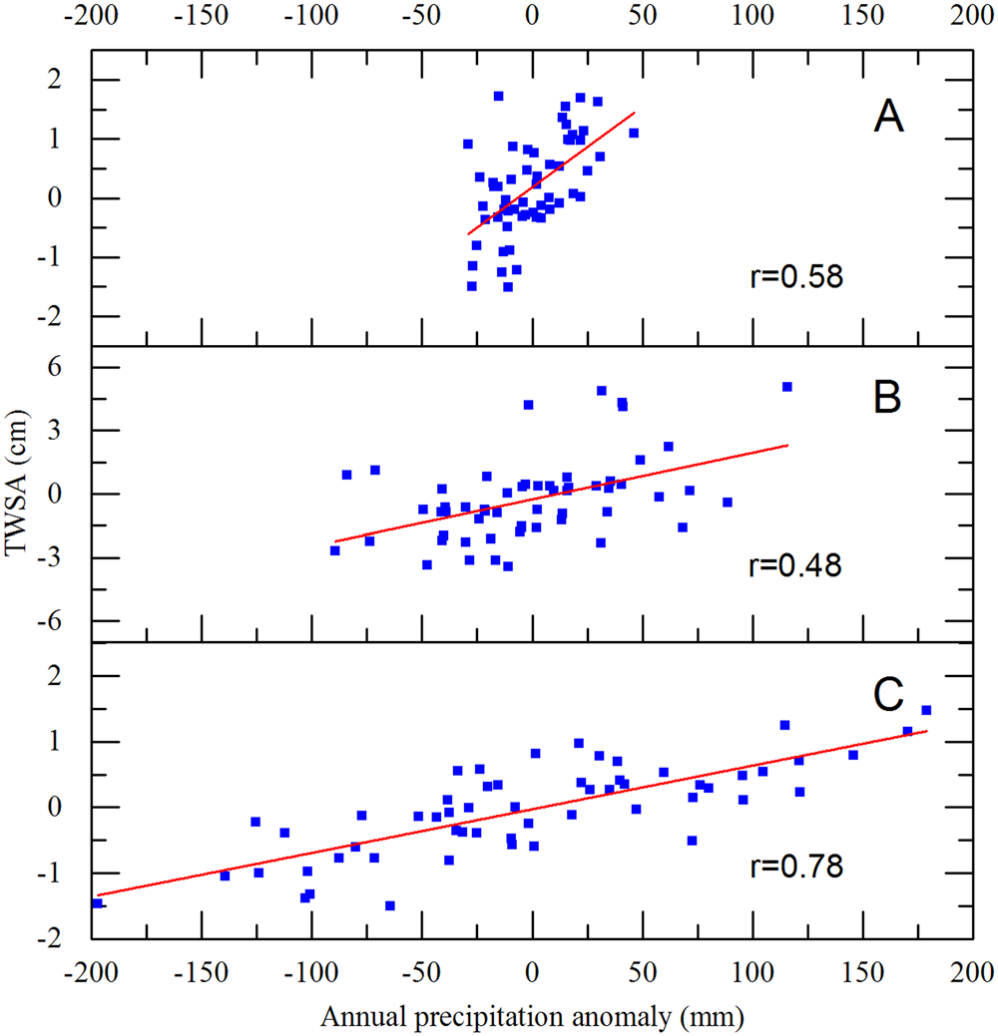
The correlation between the annual precipitation anomaly and TWSA of each sub-region (**A,B** and **C**). The red line is the simple linear regression fitting line (*p* < 0.01). This result was performed using Origin 9.0 software.

In this paper, TWS is the sum of soil moisture, canopy water storage and snow water equivalent^19^. It is influenced by precipitation and anthropogenic activities^20^. Precipitation is one of the major recharge sources to water storage. The results suggested that TWS variations in each sub-region were significantly influenced by precipitation (Fig. 6). In the insignificant change region (sub-region C), the TWSA change was highly positively correlated to precipitation (r = 0.78), and the variation patterns were consistent, whereas the TWSA changes showed lower positive correlations with precipitation in the other two regions (r = 0.58 in sub-region A and r = 0.48 in sub-region B). This suggested that water storage variability was mainly affected by precipitation in southern China, whereas multiple factors, including precipitation, jointly influenced water storage variations in other parts of China from 1948 to 2015.

### Factors impacting TWSA variability in sub-region A

In sub-region A, snow water equivalent is an important component to TWS. Precipitation and glacier melting are the major recharge sources for water resources in sub-region A^21^. This region has a temperate continental climate and plateau-climate with little precipitation. Glacier melting is extremely important to the hydrologic cycle^5, 22^. In the Tibetan Plateau, global climate change has affected the cryosphere^23^. Over the past decades, temperature and precipitation have both increased^24, 25^, leading to higher rates of glacier melting under this climate condition, which resulted an increase in the number of glacial lakes^26, 27^. Lei *et al*. (2013) suggested that climate change and glacier mass loss are the main factors impacting TWS variation^28^. In this paper, TWS increased significantly from the 1960s to 2015 in TQ and exhibited a significantly positive correlation to precipitation (Fig. S6). In addition, glacier melting and precipitation are also the main sources of recharge in Xinjiang province^29, 30^. TWS rose from 1960 to 2015 and the water storage change was significantly positively correlated to precipitation (Fig. S6). In the last fifteen years, TWS declined obviously, which may be caused by the slight decrease in precipitation and increased in temperature^5, 15^, resulting in more evaporation. Therefore, we suggest that climate change is the main factor influencing the water storage change in sub-region A.

### Factors impacting TWSA variability in sub-region B

The water storage anomaly declined from 1948 to 2015 in sub-region B (Fig. 3) and had a significant, but lower, positive correlation to precipitation (r = 0.48, Fig. 6). Therefore, anthropogenic activities and land cover change may be additional factors influencing the water storage change. Since the land surface propertied were changed in the process of anthropogenic activities, soil moisture varied with the land surface change. Due to population growth and rapid urbanization, wetlands have become severely degraded in China over the past decades, especially in Heilongjian Province^31, 32^. From 1955 to 1980, the amount of marshland covering the Sanjiang Plain decreased significantly as it was converted to farmland^32^. In this process, water in marshes was drained and the warming and drying trend of climate exacerbated the water storage reduction^33, 34^. Thus, climate change and marsh degradation were the main factors in the water storage decline in HLJ.

Urban expansion leads to less water vapour and more mixing of water vapour in the boundary layer, resulting in the reduction of precipitation^35^. In addition, infiltration decreased because of the low permeability of impervious surfaces. In the last 30 years, urban areas have expanded rapidly in the Beijing-Tianjin-Heibei (Jing-Jin-Ji) region and the area of impervious surface increased substantially^36, 37^, resulting in the decrease of soil moisture. Moreover, rapid population growth also leads to more water consumption during this period. We employed nighttime light data collected by DMSP/OLS to study the correlation between urbanization and TWSA variations. The water storage reduction due to factors other than evaporation was calculated by Eq. 3. The result showed that the TWSA change had a significant correlation to urbanization (r = 0.61, p < 0.01, Fig. S7), following a logarithmic relationship (R^2^ = 0.50). Therefore, we suggest that urbanization and population growth are two significant factors for the TWS decrease in HH.

### Factors impacting TWSA variability in sub-region C

Sub-region C contains almost all parts of south China, where rainfall is abundant. Unlike the sub-region A, snow water equivalent variability is not important to water storage change in sub-region C. Precipitation is the main recharge source of water storage. The high correlation between TWSA and precipitation suggests that precipitation is a relatively important control of TWSA variability. Although the water storage anomaly varied with precipitation, anthropogenic activities are not negligible. Sub-region C is one of the most densely populated parts of China and the depletion of water storage should not be ignored. In addition, the high number of dams influences the hydrological regime^38^. However, anthropogenic activities have lower influence to water storage changes in the sub-region C.

Overall, TWS variation exhibits a high correlation to climate change and land cover and land use changes. TWSA variations in different regions of China depend on the certain climate condition and geographical characteristics (land cover change and regional development plan).

## Conclusions

Water storage distributed unevenly over China and it is important to detect variation characteristics of different regions. Spatial and temporal patterns of Variations in TWS over China had been studied with GLDAS-2 Noah outputs in a long time series and divided spatial patterns of TWSA in China into three sub-regions with different variation characteristics firstly. The results revealed that water storage fluctuated significantly in a year and reached to the maximum in August. TWS tended to decrease in a long term period with the rate of 0.1 cm/yr and 59.5% area of China had decreased and 40.5% area of China tended to increase. At regional scale, changes in TWS of eight basins and four key zones had investigated. TWS of Xinjiang province and Tibet-Qinghai zones showed increased trend and TWS changes with decreased in HLJ, LH, HH, HuH and GN. We found that spatial patterns of TWSA in China can be divided into three distinct sub-regions of TWS variations with increased, TWS variations with decreased and TWS variations with insignificant tendency. The northeast had decreased remarkably with rate of −0.05 cm/yr due to climate change and anthropogenic activities. Urban expansion is a non-ignorable factor to TWS reduction in Jing-Jin-Ji region (r = 0.61, p < 0.01). The west had increased significantly with the rate of 0.03 cm/yr as precipitation increased and recharged by glacier melting. The south China had insignificant trends and TWS changed with precipitation (r = 0.78, p < 0.01).

## Methods

### Data sources

The Global Land Data Assimilation System (GLDAS) was jointly developed by scientists at NASA, GSFC, NOAA and NCEP to provide terrestrial water and energy storages data. It drives four land surface models: Noah, CLM (Community Land Model), VIC (Variable Infiltration Capacity) and Mosaic, incorporating both ground and satellite based data (e.g., the global land cover and soil type dataset)^39^. The model output produced by the Noah land surface model includes soil moisture data (10, 30, 60 and 100 cm, from the soil surface down), snow water equivalent and canopy water storage. In this paper, we collected the monthly data simulated by Noah from NASA (http://ldas.gsfc.nasa.gov/index.php) at spatial resolutions of 0.25° × 0.25° and 1° × 1°, from 1948 to 2010 and from 2011 to 2015, respectively. The GLDAS dataset was employed to present the trend of terrestrial water storage anomaly (TWSA) variations over China. As described in the Readme document, we simulated TWS by summing up the total soil moisture, accumulated snow and plant canopy surface water and calculated the terrestrial water storage anomaly (TWSA) by subtracting the average TWS from 1948 to 2010 (the dataset with a spatial resolution of 0.25° × 0.25°) and the average TWS from 2011 to 2015(the dataset with a spatial resolution of 1° × 1°), respectively.

The Gravity Recovery and Climate Experiment (GRACE) mission was launched by NASA and the Germany Aerospace Centre in 2002, to provide global mass change information by detecting gravity field changes^6^. The TWS anomaly data are provided by CSR, GFZ and JPL. We collected the monthly GRACE data from the CSR (Center for Space Research), GFZ (GeoForschungsZentrum) and JPL (Jet Propulsion Laboratory) and processed the data with a Gaussian filter. The GRACE TWSA dataset expresses TWSAs with equivalent water column height at a spatial resolution of 1° × 1°, covering the period of 2003 to 2013. We calculated the average of the TWSAs derived from different institutions to validate the TWSA derived from GLDAS.

The daily precipitation data set was collected from the National Climate Center (NCC) of the China Meteorological Administration (CMA) to explore the relationship between TWS variation and climate change. The daily precipitation data set covering the years from 1960 to 2015 was provided from 824 national meteorological stations in China. We summed up the valid precipitation data of each month as the monthly precipitation value and spatially interpolated the data to 0.25° × 0.25° using the Kriging interpolation method.

### Theil-Sen median trend analysis

The Theil-Sen median trend analysis method is a robust trend statistical method used to calculate the median slopes between all n(n-1)/2 pair-wise combinations of time series data^40^. It was used to detect the trend of TWSA variations of China and key zones from 1948 to 2010. It is calculated by:

1 $${S}_{TWS}=median(\frac{TW{S}_{j}-TW{S}_{i}}{j-i}),1948\le i < j\le 2010$$

where *S*_*TWS*_ refers to the T-S median, and *TWS*_*i*_ and *TWS*_*j*_ represent the TWS values of years *i* and *j*. If *S*_*TWS*_ > 0, TWS presents an increasing trend; otherwise, there is a decreasing trend. The Mann-Kendall test method was used to measure the significance of the trend. Results with p < 0.05 were considered significant. The trend analysis was calculated with Matlab R2013b.

### The Pearson correlation coefficient

The Pearson correlation coefficient was widely used to measure the linear correlation between two variables, giving a value between 1 and −1. If the value is less than 0, this implies a negative correlation between the two variables; otherwise, a positive correlation exists. This study used the Pearson correlation coefficient to measure the linear correlation between the TWSA variation and driving factors. The Pearson correlation coefficient is calculated by the following formula:

2 $${r}_{xy}=\frac{\sum _{i=1}^{n}({x}_{i}-\overline{x})({y}_{i}-\overline{y})}{\sqrt{\sum _{i=1}^{n}{({x}_{i}-\overline{x})}^{2}\sum _{i=1}^{n}{({y}_{i}-\overline{y})}^{2}}}$$

where *r*_*xy*_ is the simple linear correlation coefficient between the TWSA variation and driving factors; *x*_*i*_ is the TWSA or ΔW of the *i*th year; *y*_*i*_ is the precipitation anomaly or night time light index (NLI) of the *i*th year. $\overline{x}$ is the average TWSA or ΔW for all years; $\overline{y}$ is the average precipitation anomaly or NLI. Results with p < 0.05 were considered significant.

### The uncertain reduced water storage

Generally, TWSA can be inferred by the water balance with precipitation, evapotranspiration and runoff. In this study, we insist that the influence of anthropogenic activities (urbanization) to water storage change should be measured. Thus, we considered that the difference between precipitation, evapotranspiration, and TWSA (Eq. 3) may be influenced by anthropogenic activities. The uncertain reduced water storage is calculated by the following formula:

3 $${\rm{\Delta }}W=P-E-TWSA$$

where ΔW is the uncertain reduced water storage (cm); P is the precipitation (cm); E is the evapotranspiration (cm).

## Electronic supplementary material

Supplementary Information

## References

[1] Fu GB (2013). Temporal variation of extreme rainfall events in China, 1961-2009. Journal of Hydrology.

[2] Liu ZY (2013). Spatiotemporal characteristics of dryness/ wetness conditions across Qinghai Province, Northwest China. Agricultural and Forest Meteorology.

[3] Sheffield J (2009). Closing the terrestrial water budget from satellite remote sensing. Geophysical Research Letters.

[4] Awange JL (2014). Water storage changes and climate variability within the Nile Basin between 2002 and 2011. Advances in Water Resources.

[5] Yang P, Chen YN (2015). An analysis of terrestrial water storage variations from GRACE and GLDAS: The Tianshan Mountains and its adjacent areas, central Asia. Quaternary International.

[6] Ndehedehe C (2016). Understanding changes in terrestrial water storage over West Africa between 2002 and 2014. Advances in Water Resources.

[7] Frappart F, Seoane L, Ramillien G (2013). Validation of GRACE-derived terrestrial water storage from a regional approach over South America. Remote Sensing of Environment.

[8] Long D (2017). Global analysis of spatiotemporal variability in merged total water storage changes using multiple GRACE products and global hydrological models. Remote Sensing of Environment.

[9] Long D (2013). GRACE satellite monitoring of large depletion in water storage in response to the 2011 drought in Texas. Geophysical Research Letters.

[10] Yang T, Wang C, Yu ZB, Xu F (2013). Characterization of spatio-temporal patterns for various GRACE- and GLDAS-born estimates for changes of global terrestrial water storage. Global and Planetary Change.

[11] Syed TH (2008). Analysisi of terrestrial water storage changes from GRACE and GLDAS. Water Resources Research.

[12] Song CQ, Huang B, Ke LH (2013). Modeling and analysis of lake water storage changes on the Tibetan Plateau using multi-mission satellite data. Remote Sensing of Environment.

[13] Jiao JJ, Zhang XT, Wang XS (2015). Satellite-based estimates of groundwater depletion in the Badain Jaran Desert, China. Scientific Reports.

[14] Huang Y (2013). Analysis of long-term terrestrial water storage variations in the Yangtze River basin. Hydrology and Earth System Sciences.

[15] Yang T (2015). Climate change and water storage variability over an arid endorheic region. Journal of Hydrology.

[16] Zhang ZZ, Chao BF, Chen JL, Wilson CR (2015). Terrestrial water storage anomalies of Yangtze River Basin droughts observed by GRACE and connections with ENSO. Global and Planetary Change.

[17] Xie ZY (2016). Spatial partitioning and temporal evolution of Australia’s total water storage under extreme hydroclimatic impacts. Remote Sensing of Environment.

[18] Long D (2014). Drought and flood monitoring for a large karst plateau in Southwest China using extended GRACE data. Remote Sensing of Environment.

[19] Mo X, Wu JJ, Wang Q, Zhou H (2016). Variations in water storage in China over recent decades from GRACE observations and GLDAS. Natural Hazards & Earth System Sciences.

[20] Reager JT, Thomas BF, Famiglietti JS (2014). River basin flood potential inferred using GRACE gravity observations at several months lead time. Nature Geoscience.

[21] Li, Z. Q., Li, K. M. & Wang, L. Study on recent glacier changes and their impact on water resources in Xinjiang, north western China. Quaternary Sciences, **30**, 96–106, doi:10.3969/j.issn.1001-7410.2010.01.09 (2010). (in Chinese)

[22] Peters J, Bolch T, Gafurov A, Prechtel N (2015). Snow cover distribution in the Aksu catchment (central Tien Shan)1986-2013 based on AVHRR and MODIS data. IEEE Journal of Selected Topics in Applied Earth Observations and Remote Sensing.

[23] Zhang B (2011). Estimation and trend detection of water storage at Nam Co Lake, central Tibetan Plateau. Journal of Hydrology.

[24] Zhang B (2013). Monitoring changes of snow cover, lake and vegetation phenology in Nam Co Lake Basin (Tibetan Plateau) using remote sensing (2000–2009). Journal of Great Lakes Research.

[25] Yang K (2014). Recent climate changes over the Tibetan Plateau and their impacts on energy and water cycles: A review. Global and Planetary Change.

[26] Phan VH, Lindenbergh R, Menenti M (2012). ICESat derived elevation changes of Tibetan lakes between 2003 and 2009. International Journal of Applied Earth Observation and Geoinformation.

[27] Wang X, Siegert F, Zhou AG, Franke J (2013). Glacier and glacial lake changes and their relationship in the context of climate change, Central Tibetan Plateau 1972-2010. Global and Planetary Change.

[28] Lei YN (2013). Coherent lake growth on the central Tibetan Plateau since the 1970s: characterization and attribution. Journal of Hydrology.

[29] Lyu JQ, Shen B, Li HE (2015). Dynamics of major hydro-climatic variables in the headwater catchment of the Tarim River Basin, Xinjiang, China. Quaternary International.

[30] Ke CQ (2016). Variability in snow cover phenology in China from 1952 to 2010. Hydrology and Earth System Sciences Discussions.

[31] Niu ZG (2012). Mapping wetland changes in China between 1978-2008. Chinese Science Bulletin.

[32] Gong P (2010). China’s wetland change (1990-2000) determined by remote sensing. Science China Earth Sciences.

[33] Liu JP, Sheng LX, Lu XG, Liu Y (2015). A dynamic change map of marshes in the Small Sanjiang Plain, Heilongjiang, China, from1955 to 2005. Wetlands Ecology and Management.

[34] Gao J, Liu YS (2011). Climate warming and land use change in Heilongjiang Province, Northeast China. Applied Geography.

[35] Zhang CL (2009). Impacts of urban expansion and future green planting on summer precipitation in Beijing metropolitan area. Journal of Geophysical Research.

[36] Zhang ZX (2016). A comparative study of urban expansion in Beijing, Tianjin and Tangshan from the 1970s to 2013. Remote Sensing.

[37] Wu WJ, Zhao SQ, Zhu C, Jiang JL (2015). A comparative study of urban expansion in Beijing, Tianjin and Shijiazhuang over the past three decades. Landscape and Urban Planning.

[38] Mei X, Dai Z, van Gelder p, Gao J (2015). Linking Three Gorges Dam and downstream hydrological regimes along the Yangtze River, China. Earth and space science.

[39] Rodell M (2004). The Global Land Data Assimilation System. Bulletin of the American Meteorological Society.

[40] Jiang WG (2015). Spatio-temporal analysis of vegetation variation in the Yellow River Basin. Ecological Indicators.

