# Supplementary material for: Detection of the spatial patterns of water storage variation over China in recent 70 years
Source: Sci Rep. 2017 Jul 25;7:6423. doi: 10.1038/s41598-017-06558-5 (PMC5526944; doi:10.1038/s41598-017-06558-5)
Supplement: Supplementary file 1 — Supplementary Information [file 41598_2017_6558_MOESM1_ESM.pdf]

Supplementary Information for:

Detection of the spatial patterns of water storage variation over China in recent 70 years

Zheng Chen[1, 2], Weiguo Jiang[1,2], Jianjun Wu[1,2], Kun Chen[3], Yue Deng[1,2], Kai Jia[1,2] & Xinyu Mo[4]

[1] Key Laboratory of Environmental Change and Natural Disaster, Beijing Normal University, Beijing 100875, China. [2] Faculty of Geographical Science, Beijing Normal University, Beijing 100875, China. [3] School of Resources and Environmental Engineering, Ludong University, Yantai 264025, China. [4] State Key Laboratory of Remote Sensing Science, Institute of Remote Sensing and Digital Earth, Chinese Academy of Sciences, Beijing 100101, China.

Corresponding author

Weiguo Jiang

Key Laboratory of Environmental Change and Natural Disaster,

Beijing Normal University,

Beijing,

China

Email: jiangweiguo@bnu.edu.cn

Tel: +86 10 58809318

Fax: +86 10 58809318

Supplementary Figures

We compared the monthly TWSA derived from GLDAS with that derived from GRACE over China since 2003 to confirm whether the GLDAS data could explain water storage variations in China. As shown in Fig. S1, both TWSAs derived from GRACE and GLDAS showed significant seasonal variable characteristics, reaching to maximum and minimum values almost simultaneously; however, the amplitude of the TWSA derived from GLDAS is smaller than that of the TWSA derived from GRACE. The reason for this difference is that the TWSA derived from GRACE includes ground water and deep subsoil water that cannot be derived from GLDAS. Over all, the two TWSA datasets agree well, with a coefficient of determination (R^2) of 0.73. This suggests that the TWSA derived from GLDAS could be used to explore the water storage change pattern of China in a long time series.

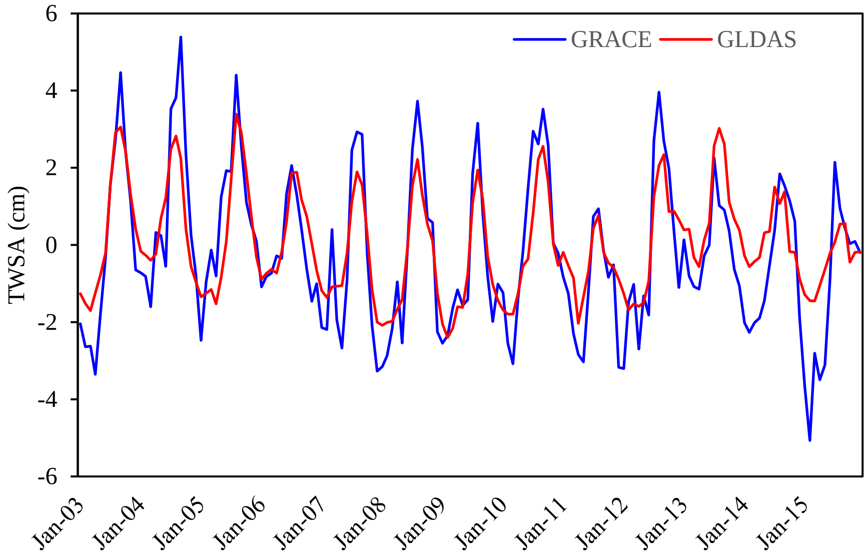

Fig. S1. The time series of monthly average TWSA obtained from GLDAS (the blue line) and GRACE (the red line) over China for the period of January 2003 to December 2015.

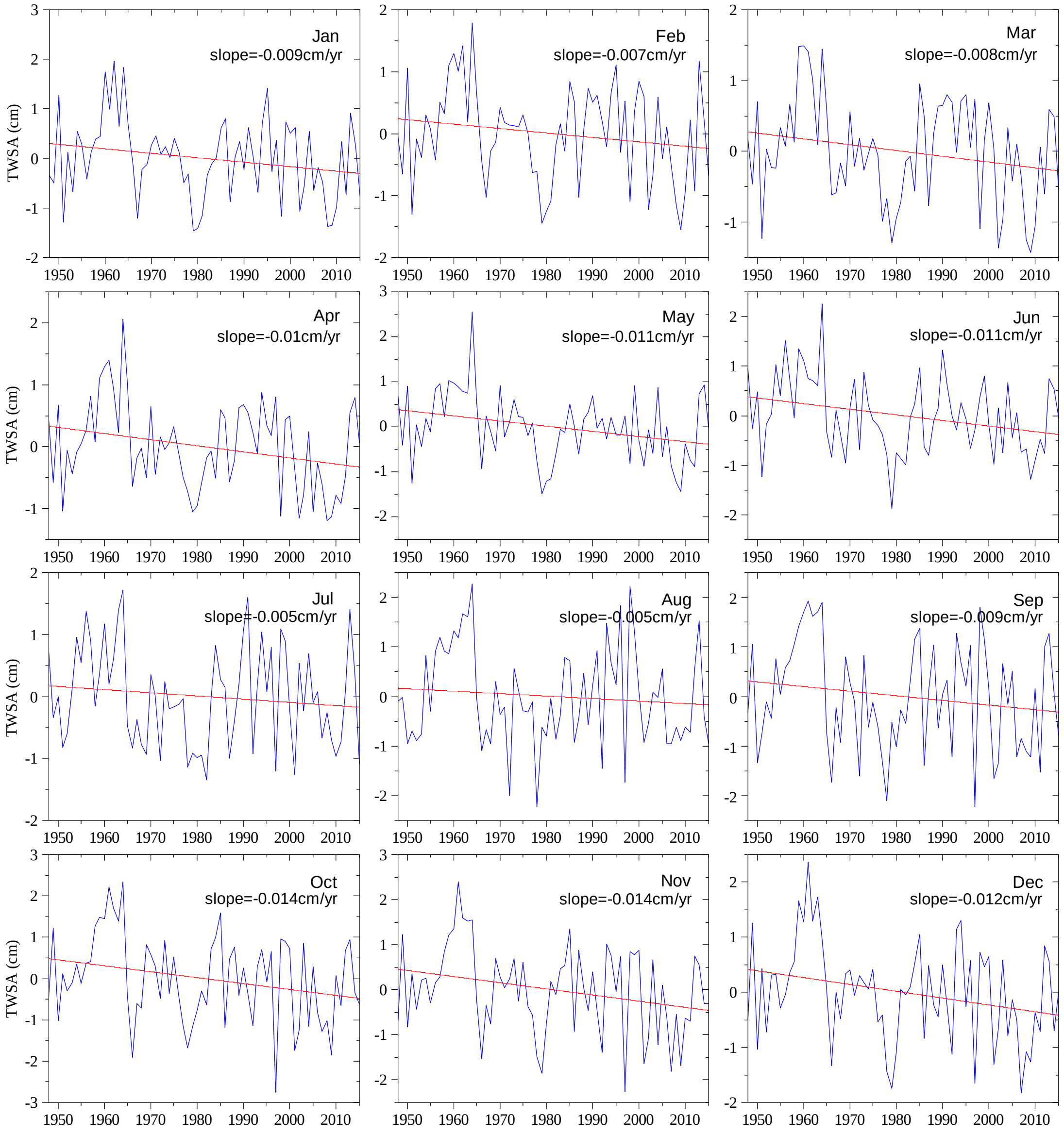

Fig. S2. The long term monthly TWSA variability from 1948 to 2015. The red line is the linear regression curve. This result was performed using Origin 9.0 software.

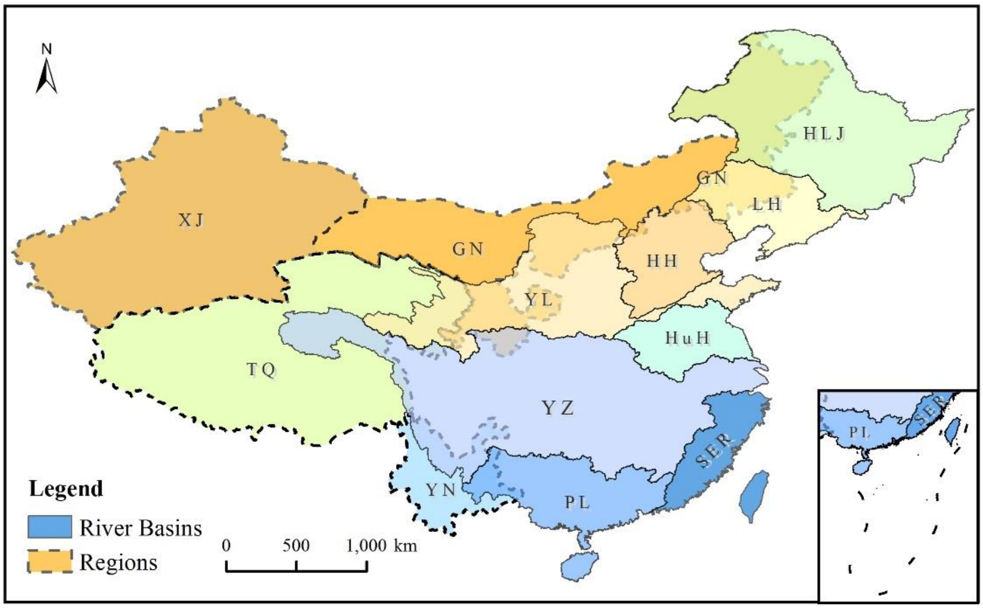

Fig. S3. The spatial distribution of key zones. This map was created using ArcGIS 10.1 software, visit http://desktop.arcgis.com/en/ for more details.

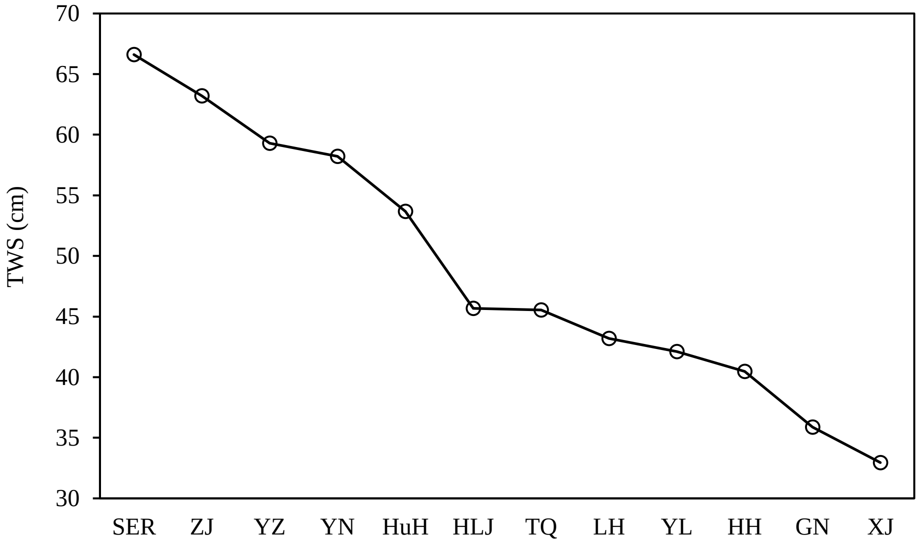

Fig. S4. The average annual TWS of each key zone from 1948 to 2015.

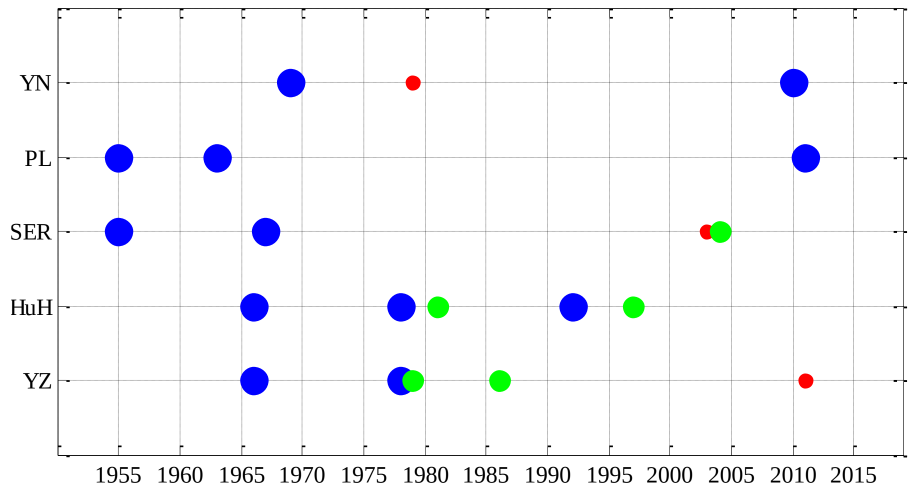

Fig. S5. Droughts occurred in YZ, HuH, SER, PL and YN when the TWS show minimum values. Blue points: heavy drought; green points: moderate drought; red points: severe drought. This result was performed by MATLAB 2013b.

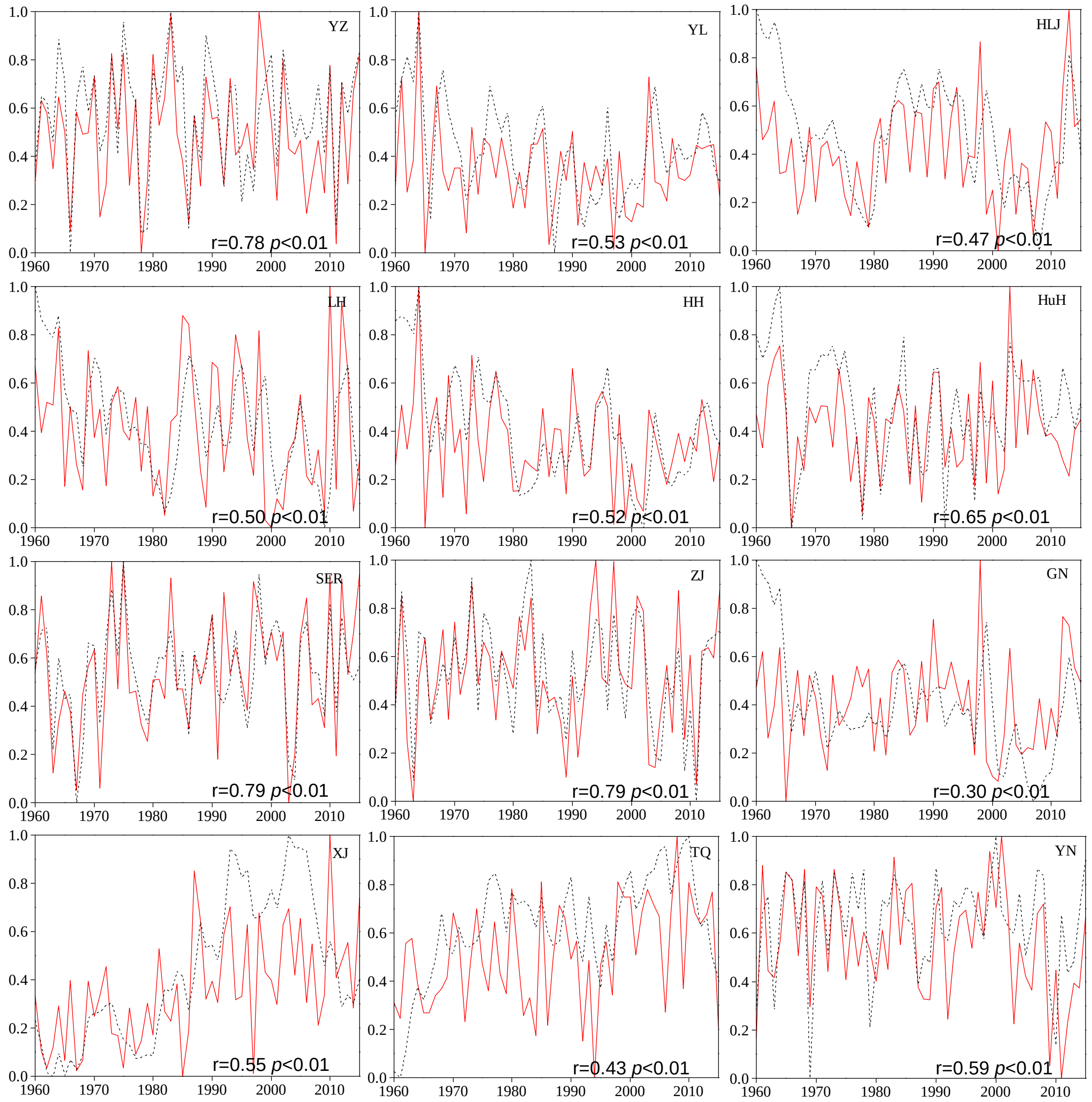

Fig. S6. Precipitation anomaly and TWSA variations in each key zone from 1960-2015. Both the

precipitation anomaly and TWSA were normalized to 0 to 1. The red line represents the precipitation change and the black line denotes the TWSA change. This result was performed using Origin 9.0 software.

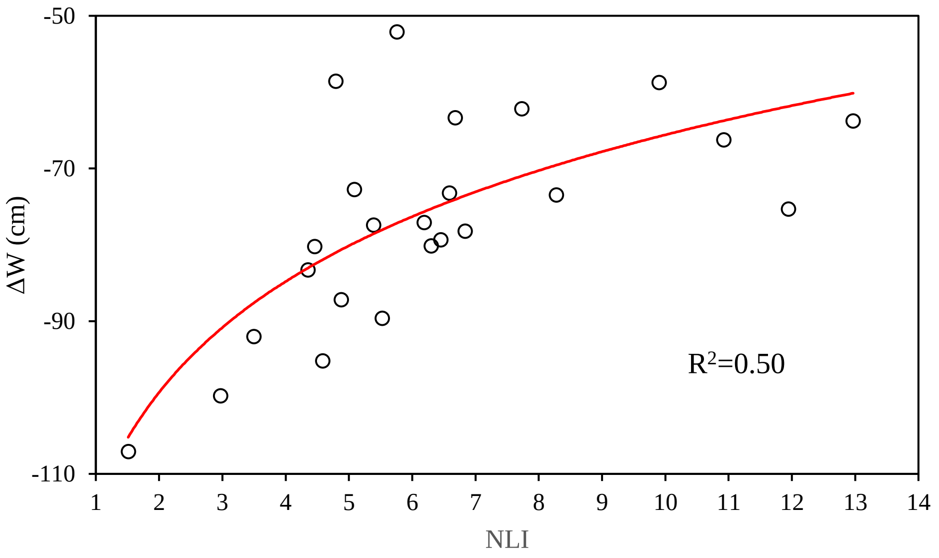

Fig. S7. The correlation between ΔW and the night time light index (NLI) in the Jing-Jin-Ji region. The time range is from 1992 to 2015.

Supplementary Tables

Table S1 Annual average TWS in key zones (cm)

Key zones	YZ	YL	HLJ	LH	HH	HuH
Annual-average	59.30	42.10	45.67	43.18	40.49	53.66
Key zones	SER	PL	GN	XJ	TQ	YN
Annual-average	66.61	63.20	35.87	32.95	45.56	58.22

Table S2 The rate of TWSA variability in key zones (cm/yr)

Key zones	YZ	YL	HLJ	LH	HH	HuH
Rate of variability	**↓0.004**	**↓0.026**	**↓0.092**	**↓0.075**	**↓0.106**	**↓0.028**
Key zones	SER	PL	GN	XJ	TQ	YN
Rate of variability	**↓0.002**	**↓0.003**	**↑0.042**	**↑0.046**	**↑0.023**	**↑0.005**
